# DNA Methylation Changes in Fibromyalgia Suggest the Role of the Immune-Inflammatory Response and Central Sensitization

**DOI:** 10.3390/jcm10214992

**Published:** 2021-10-27

**Authors:** Maria Carla Gerra, Davide Carnevali, Paolo Ossola, Alberto González-Villar, Inge Søkilde Pedersen, Yolanda Triñanes, Claudia Donnini, Matteo Manfredini, Lars Arendt-Nielsen, Maria Teresa Carrillo-de-la-Peña

**Affiliations:** 1 Center for Neuroplasticity and Pain (CNAP), SMI®, Department of Health Science and Technology, Aalborg University, 9220 Aalborg, Denmark; LAN@hst.aau.dk; 2 Centre for Genomic Regulation (CRG), The Barcelona Institute of Science and Technology, 08003 Barcelona, Spain; davide.carnevali@crg.eu; 3 Department of Chemistry, Life Sciences, and Environmental Sustainability, University of Parma, Parco Area delle Scienze 11A, 43124 Parma, Italy; claudia.donnini@unipr.it (C.D.); matteo.manfredini@unipr.it (M.M.); 4 Department of Medicine and Surgery, University of Parma, 43126 Parma, Italy; paolo.ossola@unipr.it (P.O.); mteresa.carrillo@usc.es (M.T.C.-d.-l.-P.); 5 Psychological Neuroscience Lab, Psychology Research Centre, School of Psychology, University of Minho, 4710-057 Braga, Portugal; albertojac.gonzalez@gmail.com; 6 Department of Clinical Medicine, Aalborg University, 9000 Aalborg, Denmark; isp@rn.dk; 7 Molecular Diagnostics, Aalborg University Hospital, 9100 Aalborg, Denmark; 8 Department of Clinical Psychology and Psychobiology, University of Santiago de Compostela, 15782 Santiago de Compostela, Spain; avalia-t3@sergas.es

**Keywords:** fibromyalgia, epigenetics, blood, biomarkers, DNA methylation, depression, immune system, pain management

## Abstract

Fibromyalgia (FM) has been explained as a result of gene-environment interactions. The present study aims to verify DNA methylation differences in eleven candidate genome regions previously associated to FM, evaluating DNA methylation patterns as potential disease biomarkers. DNA methylation was analyzed through bisulfite sequencing, comparing 42 FM women and their 42 healthy sisters. The associations between the level of methylation in these regions were further explored through a network analysis. Lastly, a logistic regression model investigated the regions potentially associated with FM, when controlling for sociodemographic variables and depressive symptoms. The analysis highlighted significant differences in the *GCSAML* region methylation between patients and controls. Moreover, seventeen single CpGs, belonging to other genes, were significantly different, however, only one cytosine related to *GCSAML* survived the correction for multiple comparisons. The network structure of methylation sites was different for each group; *GRM2* methylation represented a central node only for FM patients. Logistic regression revealed that depressive symptoms and DNA methylation in the *GRM2* region were significantly associated with FM risk. Our study encourages better exploration of *GCSAML* and *GRM2* functions and their possible role in FM affecting immune, inflammatory response, and central sensitization of pain.

## 1. Introduction

Fibromyalgia (FM) is a long-term pain syndrome characterized by chronic widespread pain (CWP) and a constellation of additional comorbidities, mainly including fatigue, sleep impairment, depression, and cognitive dysfunctions. Its prevalence is 2–4% in the general population, with higher frequencies in women than in men [1]. Thanks to the development of diagnostic criteria [2], and the recently approved ICD-11 (International Classification of Diseases 11th Revision) coding system [3], the diagnosis of FM has improved in the last years. However, one of the main problems remains the lack of objective markers.

The pathogenesis of this disease is unclear. Central sensitization, with alterations in nociceptors, neurons, and glia processing pain signals, was proposed to explain CWP [4]; consistently, FM has recently been characterized as nociplastic pain according to a mechanistic description [5]. Other hypotheses for FM pathogenesis include altered inflammatory mechanisms and altered immune system response [6,7], abnormalities of hypothalamic-pituitary-adrenal (HPA) axis [8], or altered dopamine response to pain [9].

A gene-environment interaction, predisposing or protecting against FM risk, model seems to be the best explanation for these pathological phenotypes. The combinations of polymorphisms in the serotoninergic and catecholaminergic pathways [10,11] and environmental factors [12] might be mediated or affected by epigenetic changes. Epigenetics includes heritable changes in the gene function that cannot be explained by changes in the DNA sequence, influencing both gene expression and phenotype [13,14]. In particular, DNA methylation is an epigenetic mark involved in gene expression regulation, catalyzed by a family of DNA methyltransferases that transfer a methyl group from S-adenyl methionine onto the DNA cytosine to form 5-methylcytosine [15]. Studies proposed that DNA methylation might reflect or contribute to the complex gene-environment interplay inducing FM pathogenesis [11]. However, the processes by which epigenetics might fine-tune the relationship between the genetic background, experienced stress and the development of FM remain a major challenge, particularly in humans. The few studies evaluating DNA methylation as a potential biomarker of FM [11] often considered a heterogeneous population with incomplete phenotypic descriptions, and no attention for comorbidities that might highly impact the epigenetic signatures.

Therefore, the present study aims to explore DNA methylation in a group of FM women and their healthy sisters, subjected to extensive clinical evaluation. We used a two-fold approach. First, we compared the two groups on the methylation sites independently. Second, to better understand the complex disease state, we compared siblings’ methylation using a network-based modeling, an undirected graph holding vertices as methylation sites and edges as the association between them. Because sociodemographic and clinical variables might affect DNA methylation, we also tested the simultaneous influence of DNA methylation levels and these variables on FM development. Exploring epigenetic variations could reveal new insights in FM pathogenesis or biomarkers of the disease.

## 2. Materials and Methods

### 2.1. Subjects

Eighty-four Caucasian participants (42 female patients with FM and 42 related healthy sisters) were selected for the present study from a cohort of 543 families in which at least one member was diagnosed with FM. The more severely FM affected patients (group 5, previously described in a pilot study [16]) were recruited and diagnosed by a primary care physician or by a professional specialist in rheumatology or neurology, according to the ACR (American College of Rheumatology) 2010 criteria. For each FM patient, a related healthy sister was selected as control. The study design is shown in Figure S1.

### 2.2. Demographic and Clinical Assessment

All the participants, subjects and healthy controls (HCs), were submitted to a clinical interview about demographic data and to the following scales and questionnaires (Table S1): Fibromyalgia Impact Questionnaire (FIQ) [17,18] and Visual Analog Scales (VAS) to assess the core symptoms of FM [19]; Beck Depression Inventory (BDI) [20,21] and Pittsburgh Sleep Quality Inventory (PSQI) [22,23] to assess, respectively, depressive symptoms and sleep disturbance.

### 2.3. Samples Collection

As previously reported [16], peripheral whole blood collection, two tubes of 10 mL per subject, was performed via venipuncture and leukocytes were separated through a washing protocol. DNA purification protocol from the isolated leukocytes was performed using QIAamp DNA Blood Midi/Maxi Kit (Spin Protocol, QIAGEN) at the Galician Public Foundation of Genomic Medicine of the University of Santiago de Compostela (Spain). Aliquots of the genomic DNA extracted were sent to Aalborg University for the present epigenetic study.

### 2.4. DNA Methylation Analysis

We analyzed DNA methylation level in eleven genomic regions (Table 1), including CpGs islands, promoters, and transcription start sites. These regions of interest were representative of the main symptoms and hypotheses associated to FM pathogenesis (pain perception, inflammatory response, stress and immune system, dopaminergic pathway), as identified in a previous pilot study [16].

Targeted NextGen Bisulfite Sequencing was conducted by EpigenDx, Inc. (Hopkinton, MA, USA) in four main steps. (i) Bisulfite conversion: extracted DNA samples (500 ng) were bisulfite modified using Zymo EZ-96 DNA Methylation™ Kit (Zymoresearch, CA, USA); (ii) PCR amplification: the bisulfite-treated DNA were subsequently amplified with separate multiplex or simplex PCRs using Qiagen HotStarTaq (0.5 units), 0.2 μM primers, and 3 μL of in a final volume of 20 μL. Quality and quantity of the PCR products were checked using the QIAxcel Advanced System, and then they were pooled and purified using QIAquick PCR Purification Kit columns (Qiagen); (iii) libraries preparation: a custom Library Preparation method created by EpigenDx was used and then library molecules were purified using Agencourt AMPure XP beads (Beckman Coulter, Brea, CA, USA) and quantified using the Qiagen QIAxcel Advanced System. Barcoded samples were then pooled in an equimolar fashion before template preparation and enrichment were performed on the Ion Chef™ system (Thermo Fisher, Waltham, MA, USA) using Ion 520™ and Ion 530™ ExT Chef reagents; (iv) sequencing: following this, enriched, template-positive library molecules were then sequenced on the Ion S5™ sequencer using an Ion 530™ sequencing chip (Thermo Fisher, Waltham, MA, USA).

### 2.5. Bioinformatic Data Processing

Bisulfite treated single-end sequencing reads were mapped to the human reference genome (GRCh38/hg38), using BS-Seeker2 [38] with default parameters and bowtie2 aligner [39]. Conversion of the alignment files to the CGmap format has been performed using CGmapTools [40]; finally, Metilene [41] was used for the identification of differentially methylated regions and cytosines.

### 2.6. Statistical Analyses

Metilene software, with a binary segmentation algorithm combined with a two-dimensional statistical test [41], was used for the detection of differentially methylated regions (DMRs, test’s parameters: -f 2 -m 1 -d 0.01) using the whole-genome methylation matrix and supplying the genomic coordinates of our regions of interest. To call differentially methylated cytosines (DMCs), Metilene was supplied with a subset of the whole-genome methylation matrix containing only our regions of interest (test parameters: -f 3 -m 1 -d 0.01). In particular, the software assesses the statistical significance of potential DMRs by a two-dimensional version of the Kolmogorov–Smirnov test (KS-test) [42] and an independent Mann–Whitney U test (MWU-test). The software Metilene assesses each cytosine for differential methylation (DMCs test) by using the Mann–Whitney-U test. The corresponding *p*-values are reported in the output.

The associations between the different methylated genome regions have been explored through a network analysis. This approach aims to clarify the structure of a large number of connections that otherwise would seem irrelevant or too complicated. Specifically, being interested in the differences between the two groups (i.e., FM patients and HCs), two networks were built using partial correlations. A network is a graphical representation of the relationships (named edges) between variables (named nodes). Each network was first compared by the centrality measures and then the two networks’ structures were compared using the edge correlations. This approach defines in each network the magnitude of association between variables (edge weights). A significant correlation between these two sets of variables suggests a similarity between the two networks. The networks have been plotted with JASP (v 0.11.1.0), whereas the comparison analyses were performed using R (v 3.6.2).

Concerning the socio-demographic data and other information such as medication, the two samples were analyzed using T-tests or chi-squared tests, depending on whether they were continuous or categorical variables. The descriptive statistics for all the variables included mean, standard deviation, frequencies, and percentages.

Subsequently, a logistic regression model was estimated to test the concurrent effect of mean methylation levels in the candidate genome regions on the risk to develop FM. As the presence of depression in FM patients is a potential confounding factor, depression scores measured through BDI were also included in the model. In addition, because socio demographic variables might affect the methylation levels, the model also controlled for the following socio-demographic variables: age in years, BMI in Kg/m^2^; job status (coded as 0 = unemployed, 1 = employed); education (coded as 0 = none, 1 = primary, 2 = secondary, 3 = professional and 4 = university); living status (coded as 0 = alone and 1 = with someone). Additionally, current use of the following medications was coded as 0 = none and 1 = at least one: GABAergic medications (such as benzodiazepines), gastrointestinal medications (such as proton pump inhibitors), cardio-vascular medications (such as statins), and antihistaminic medications. Antidepressant medications and painkillers were not included because they represent the gold-standard treatment for FM and hence would have perfectly split the two samples. Since the sample size did not allow the inclusion of all the co-variates, a two-step Cluster Analysis (including both continuous and categorical variables) was applied to reduce the dimensionality of the covariates (IBM SPSS 26.0). The distance between the variables was the log-likelihood. The number of clusters extracted was automatic and based on the Bayesian Information Criterion (BIC).

The clusters have been thus added in the regression model with the candidate genome regions and depression.

Robust standard errors were applied to the regression models in order to reduce the possible bias introduced in the estimations by heteroscedasticity. The regression analyses were conducted in Stata/IC 15.1 (StataCorp, College Station, TX USA).

For all the statistical analyses, results were considered statistically significant for *p* ≤ 0.05.

## 3. Results

### 3.1. Characteristics of the Study Participants

The forty-two FM women were aged 22–75 years (mean age 50 ± 10 years) and the 42 related healthy sisters were aged 28–72 years (mean age 47 ± 10 years). Table 2 shows the patients’ characteristics. Questionnaires’ and scales’ scores related to depression (BDI), sleep impairment (PSQI), and the main FM symptoms of pain (FIQ, WPI, SSS, VAS) were all significantly higher in patients than controls (*p* < 0.000). Among the other variables, the number of participants consuming anti-anxiety, antidepressant, anti-inflammatory medications, and opioids painkillers were significantly higher in FM women compared with controls (*p* < 0.000). The average time since diagnosis was about 9 years, reflecting the long-term course of FM management.

### 3.2. DNA Methylation Analysis Comparing Cases and Controls

Two types of analyses were applied: the differentially methylated regions (DMRs) test to determine methylation by grouping neighboring cytosines, and the differentially methylated cytosines (DMCs) test to reveal methylation at single cytosine level (Table S2).

Concerning DMRs (Table 3), only one differentially methylated region (methylation difference ≥ 1%), evidenced by Metilene software, showed significant differences by using Mann–Whitney U-test (MWU-test) and the Kolmogorov–Smirnov test (KS-test): the region (Figure S2) was related to *GCSAML* gene, the Germinal center associated signaling and motility like gene, a putative signaling protein with a potential function in the immune response. The *GCSAML* region (chr1: 247518380–247518621) showed a level of methylation significantly higher in FM patients (mean methylation 0.168) than controls (mean methylation 0.158) with both tests (*p* (MWU) = 0; *p* (KS) = 0.007).

Concerning DMCs, differences in the level of methylation in the single cytosines of the included regions comparing FM women with their healthy sisters were verified by using Mann–Whitney U-tests. The corresponding *p*-values reported in the output (Table 4) showed seventeen differentially methylated cytosines, belonging to six genes, *GCSAML, DRD3, TRPA1, IL25, OXT*, and *MCF2*, that reached statistical significance (*p* < 0.05). In particular, five cytosines were evidenced in the *GCSAML* gene (methylation difference ≥ 5.06%), confirming once again the possible correlation of this gene with FM. Three DMCs were related to the region of the *TRPA1* gene, encoding a receptor involved in pain detection (methylation difference ≥ 1.91%). Another five cytosines were evidenced in the *OXT* gene (methylation difference ≥ 1.8%), the oxytocin hormone, involved in stress, cognition, and complex behavior. Two DMCs resulted in the *MCF2* gene region (methylation difference ≥ 4.8%); this gene encodes an oncogenic protein, a member of the DBL family of Rho GEFs (Rho GDP–GTP exchange factors) that exerts control over some members of the Rho family small GTPases. Finally, one differentially methylated cytosine was identified in the *DRD3* gene (chr3: 114178637–114178638; methylation difference = 3.6%), and one in the *IL25* region (chr14: 23372248–23372249; methylation difference = 3.8%). Thirteen out of these seventeen identified cytosines were higher methylated in FM women compared with their healthy sisters. Four cytosines (*GCSAML*, chr1: 247518586–247518587; *TRPA1*, chr8: 72076406–72076407; *OXT*, chr20: 3071336–3071337/chr20: 3071468–3071469) resulted in lower methylated in FM women.

However, applying the correction for multiple comparisons, the significance of the DMRs and DMCs disappeared, except for one cytosine (chr1: 247518586–247518587) related to the *GCSAML* gene.

### 3.3. Network Analysis

**Figure 1:**
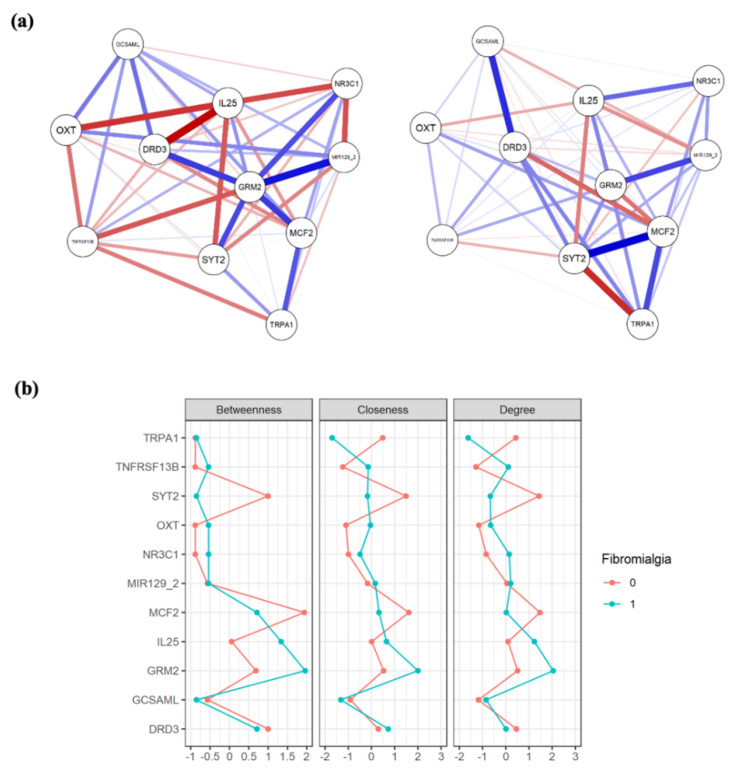
(**a**) Network graphs for subjects with FM (**left**) and their healthy sisters (**right**). The nodes represent the variables included (i.e., level of methylation in the analyzed regions), and the lines connecting the nodes (edges) represent the association between them. Positive and negative associations are blue and red colored, respectively. The strength of the associations (weight) is reflected in the thickness of the line: thicker lines correspond to stronger associations. At a visual inspection the structures resulted different, with *GRM2* having more and stronger connections in the FM sample only. (**b**) Comparison of the centrality plots of the two networks in FM patients (coded as 1, light blue) and their healthy sisters (coded as 0, orange). The betweenness quantifies the number of times a node acts as a bridge along the shortest path between two other nodes. The closeness of a node is the average length of the shortest path between the node and all other nodes in the graph. The degree is defined as the number of links incident upon a node (i.e., the number of ties that a node has). On the *y* axis the methylation sites are reported, and on the *x* axis the strength of these three dimensions (betweeness, closeness, and degree) is evidenced. Higher scores suggest a greater importance of the methylation site in the network structure. As confirmation of the inspection level, *GRM2* had greater levels of betweeness, closeness, and degree, having the highest value among all the methylation sites in the FM group only. This was not true for the healthy sisters, in which *SYT2* and *MCF2* were more central to the network.

A visual inspection evidenced that the two extracted networks (Figure 1a) showed different centrality measures (Figure 1b): for the patients, DNA methylation level in the *GRM2* region represented the most central and connected node, whereas for the HCs group, the most central node is the *MCF2* methylation level. The analysis of correlation between edges showed a non-significant correlation between the networks (r = 0.1169553; *p* = 0.3940608), confirming a different structure for each sample.

### 3.4. Cluster Analysis of the Socio-Demographic Variables

The cluster analysis extracted two clusters with a sufficient quality and balance (ratio between the two clusters: 2.23). The main three predictors of the clusters were employment, education, and GABAergic medications. The first cluster (*n* = 22, 31%) was constituted mainly by subjects that were employed (95.5%), had a university education (40.9%), were not taking benzodiazepines (95.5%) nor other medications, and were slightly younger (mean age 43.64 years). On the other hand, the subjects in the second cluster (*n* = 49; 69%) were older (mean age 52.12), mostly had only primary education (71.4%), were housewives or unemployed (67.3%), and nearly half were taking benzodiazepines (53.1%). The two clusters were almost overlapping regarding living status and BMI. When comparing the two clusters on the diagnosis of FM, these were not significantly different (chi-squared = 3.896; *p* = 0.72).

### 3.5. The Concurrent Effect of DNA Methylation, Depression, and the Clustered Sociodemographic Data on FM Risk

We performed a binary logistic regression to predict the risk to develop FM, including in the model as independent variables the mean methylation levels of the candidate genome regions, depression, and the sociodemographic clusters. The diagnosis of FM was included as a dependent variable (coded as 0 = no and 1 = yes) (Table 5). Among the methylated regions inserted in the model, only the level of methylation in *GRM2* gene was significantly and negatively associated with FM diagnosis; in particular, the DNA methylation increase in the *GRM2* region (chr3: 51706813–51707270) resulted to confer 39% lower risk to develop FM (OR = 0.614; 95% CI = 0.388–0.971; *p* = 0.037). Among the other variables entered in the model, only depressive symptoms remained associated with FM: a unit increase in BDI scale (used to measure depression) corresponded to 1.3 times higher risk of suffering from FM (OR = 1366; 95% CI = 1.170–1.594; *p* < 0.000).

It is important to note that the variables inserted in the present model resulted to account for about 56% of the total variability of the dependent variable FM.

## 4. Discussion

The present study analyzed the DNA methylation levels in eleven genome regions of 42 women with fibromyalgia compared with their 42 healthy sisters, using a siblings approach that reduces the genetic heterogeneity and differential prenatal or early-life exposures: this represents a powerful design to investigate the association of DNA methylation with FM. The comparison revealed a slight but significant different methylation level in the *GCSAML* gene region and identified differentially methylated cytosines in *GCSAML, DRD3, TRPA1, IL25, OXT*, and *MCF2* genes regions. Nevertheless, except for one cytosine in the *GCSAML* gene, all the differences disappeared, applying the correction for multiple comparison. The networks analysis revealed a significantly different structure of methylation sites comparing the two groups, with *GRM2* methylation representing a central node only in the FM group. When testing the simultaneous effects of the mean methylation levels in the candidate genome regions together with depression and the clustered sociodemographic clinical data, *GRM2* methylation was significantly and negatively associated with FM risk, while depression was positively associated with it.

### 4.1. DNA Methylation Analysis

Our findings in the differentially methylated regions (DMRs) test brings the focus on the *GCSAML* gene, with an increased methylation level observed in FM women compared with their healthy sisters. The *GCSAML* gene, which was observed to be subjected to epigenetic regulation, encodes a signaling molecule thought to be associated to the sites of proliferation and differentiation of mature B lymphocytes [28]. This result points out the connection with potential immune system dysfunction in FM patients. Previous investigations revealed altered expression of immune pathways and markers of tissue destruction in FM women [30,43,44]. In chronic fatigue syndrome, differentially methylated [45] and differentially expressed genes related to the immune response [46,47] were identified. A recent study also showed specific transposable elements overexpressed in the immune cells of FM patients [48]. Taken as a whole, these studies, together with our finding, appear to support a possible immune system dysregulation in FM. However, most of the epigenetic studies on myalgic encephalomyelitis, chronic fatigue syndrome, or chronic pain reported a hypomethylation trend [45,49], in contrast with our results, indicating possible different epigenetic regulation in FM compared with other chronic pain states.

Additionally, in the DMCs test, of the seventeen differentially methylated cytosines identified, only four were found less methylated in FM patients, while all the others were shown to be higher methylated in FM patients compared with controls, indicating the importance of the single base resolution technology as a valid approach to detect different trends in methylation within the same region. The genes evidenced in the DMCs test are related to neuronal development, dopaminergic and pain pathways, inflammation, and sociability. In particular, five differentially methylated cytosines confirmed *GCSAML* as possibly involved in FM pathogenesis. The *DRD3* and *IL25* genes, in which only one DMC was found, recall the involvement of the dopaminergic pathway and inflammation, respectively, in FM patients. Dopamine was shown to significantly influence pain perception, with striatal dopamine release associated with pain inhibition and *DRD3* Ser9Gly polymorphism related to thermal pain perception in CWP patients [34,50]. The inflammatory cytokines IL25 was previously found to be up-regulated in FM [30]. The three cytosines identified in *TRPA1* bring the focus on the results of Bell and coworkers, in which the promoter region methylation was inversely associated with both heat pain and pressure pain thresholds [26]. Two DMCs were evidenced in the proto-oncogene *MCF2*, which modulates the activity of small GTPases, and it is involved in dendrite elongation and neurite outgrowth [51]. Interestingly, *MCF2* genetic and epigenetic variants were associated with FM and many other pathological and psychiatric diseases [52]. Five significantly differentially methylated (>1%) cytosines were instead detected in the oxytocin gene. Oxytocin is relevant for the perturbations in the HPA axis observed in FM patients, because it was shown to induce adreno-corticotropin-hormone release at the anterior lobe of the pituitary [8]. In healthy subjects, oxytocin decreases cortisol release and anxiety in response to social stress [53]; its anti-nociceptive, analgesic, anxiolytic, and sedative effects are well known [54,55].

### 4.2. Concomitant Risk Factors on FM Risk

As shown by the logistic regression model, testing the simultaneous incidence of DNA methylation changes, depression, and the clustered sociodemographic data on the risk to develop FM, *GRM2* DNA methylation and depression were confirmed to increase FM risk. Interestingly, our results put the attention on the role of *GRM2* gene methylation, which was also evidenced in our previous pilot study [16], although with not consistent results. *GRM2,* encoding the Glutamate Metabotropic Receptor 2, affects glutamate release, the major excitatory neurotransmitter in the central nervous system CNS, and thus might be involved in both central sensitization and immune/inflammatory pathological mechanisms [56,57]. In addition, even though the groups comparison did not yield any significant result after controlling for multiple testing, the network analysis allowed a more fine-grained interpretation of the results. In fact, we showed how *GRM2* methylation represented a central node only in the FM sample, suggesting its relevance in the pathogenesis of the disorder. Moreover, this methylation site was not equally important in their healthy sisters that had a completely different structure. Nevertheless, the small sample size in our study and the correlational nature of the network analysis limits the generalizability of the results and does not allow inferring causality. Further modelling and investigations of networks’ structural difference between siblings are needed.

**Figure 2:**
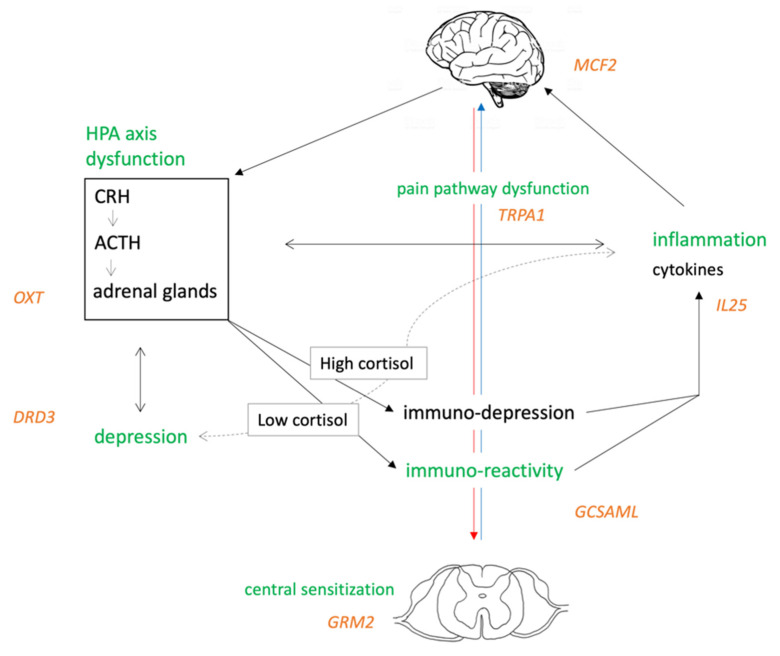
Proposal of an etiopathogenesis model for FM: the HPA axis is the primary stress response system, and it is regulated by the CNS. In addition, depression and poor sleep/fatigue development, both characterized by elevated peripheral inflammatory markers, are influenced by the HPA axis.

Our results might support the hypothesis of an altered immune system response in FM. We propose that this altered pathway, in which *GCSAML* might have a role, could be cause or consequence of the complex FM phenotype. As shown in Figure 2, the HPA axis is the primary stress response system, and its activation results in downstream production of cortisol and a dampening of the immune response [57]. FM syndrome was found to be associated with hypocortisolism [58], and low cortisol levels may be associated to immune system hyper-reactivity with subsequent activation of inflammatory markers. Peripheral inflammatory mediators have been shown to directly induce transcriptional modulation in the brain [59]. Completing this loop, the CNS, in particular the brain stem catecholaminergic centers, may in turn regulate the HPA axis [60]. In addition, the HPA axis has been implicated in the pathophysiology of depression [61], in turn associated with peripheral inflammatory markers. Unfortunately, it is not possible to establish any causal relationship among the evidenced pathways, and future longitudinal designs are encouraged to clarify the contribution of the factors involved in FM.

### 4.3. Limitations and Future Research Directions

A major strength of this study is the inclusion of biological siblings unaffected by FM as controls, but certain limitations should be highlighted. First, it is still inconclusive if the identified DNA methylation differences are mechanisms of the disease or result from a response towards environmental stimuli. Early environmental stressors can cause CpGs hypermethylation, altering the HPA axis responses to stress: considering epigenetic factors with no correlation with personal life experiences can be deeply misleading. The second limitation is related to the analyzed population: it included 84 participants and may thus be too small to detect differential DNA methylation and strong associations with the participants characteristics. Further investigation into differential methylation between FM patients and healthy controls remains necessary. In addition, only women were included, and thus the results may not be generalized to male patients with FM. Third, potential transcriptional changes related to the altered methylation were not investigated because they required higher starting material, and this should be considered in future studies. Moreover, DNA methylation in peripheral blood is not necessarily directly reflective of central pain mechanisms, but it could serve as a peripheral epigenetic biomarker, as similar levels of DNA methylation were observed in blood and brain tissues at multiple pain regions in previous studies. Replication studies using specific brain and dorsal root ganglia tissues should help to further clarify the role of DNA methylation in FM.

## 5. Conclusions

The results of the present study recall the bidirectional communication between the brain and the immune system, and they are consistent with clinical data showing a complex involvement of depression in FM pathogenesis. FM seems to be the result of a complex interplay between stress system alterations that might trigger depression and pain pathway dysfunctions. In addition, we identified *GCSAML* and *GRM2* as interesting targets that need to be considered in future research to unravel their role in FM and provide useful biomarkers to improve diagnosis and treatment for it.

## Figures and Tables

**Table 1:** List of the eleven targeted genome regions included in the DNA methylation analysis: gene, chromosome position (start-stop), previous genes’ associations with specific conditions are reported (Referred to: UCSC Genome Browser on Human December 2013 (GRCh38/hg38) Assembly).

Gene	Chr	Target Region		Relevant Association	PMID
		Start	End		
*SYT2*	chr1	202709820	202709921	Long-term changes in DNA methylation in the PFC in a chronic pain model.	25852480 [24]
*TNFRSF13B*	chr17	16971786	16972349	DNA methylation changes associated to chronic widespread musculoskeletal pain.	28221285 [25]
*TRPA1*	chr8	72075180	72076496	Differentially methylated regions associated with high or low pain sensitivity and with chronic pain.	24496475 [26] 26849948 [27]
*GCSAML*	chr1	247518380	247518621	In a DMR, maternally inherited 5mCpG imprints with potential influence on transcription factors expression from the paternal allele.	29545821 [28]
*MIR129-2*	chr11	43581119	43581338	Hypermethylation of CpG islands in the *miR-219* promoter in a chronic inflammation pain model.	25031391 [29]
*IL25*	chr14	23372249	23372369	Differentially expressed comparing FM patients and controls.	27157394 [30]
*MCF2*	chr X	139692217	139692357	DNA methylation changes in borderline personality disorder.	24367640 [31]
*GRM2*	chr3	51706813	51707270	Epigenetically regulation of type-2 metabotropic glutamate receptor in models of chronic inflammatory, neuropathic pain and visceral nociception.	28326943 [32] 25378524 [33]
*DRD3*	chr3	114178583	114179811	Ser9Gly polymorphism associated to thermal pain thresholds and noxious inhibitory controls.	19464960 [34]
*NR3C1*	chr5	143403095	143403227	Chronic stress and early life trauma associated with methylation changes.	25263804 [35] 26817950 [36]
	chr5	143404021	143404121		
*OXT*	chr20	3071310	3071744	DNA methylation (presumably linked to higher *OXT* expression) associated to sociability in humans.	27325757 [37]

**Table 2:** Comparison of the two groups on the clinical and socio-demographic variables: age, weight, height, Body Mass Index (BMI), living status, employment, number of children, level of education, years since diagnosis. The use of specific drugs and scores related to the following questionnaires are reported: BDI, PSQI, FIQ, WPI, SSS, VAS for the main FM symptoms. Standard deviation (STDEV) is indicated in parenthesis for the collected data.

	Mean FM (±St Dev), *n* = 42	Mean HCs (±St Dev), *n* = 42	t/Chi2	*p*-Value
Age (yrs)	50.359 (±9.685)	47.564 (±10.576)	−1.4390	0.1583
Weight (kg)	70.459 (±14.017)	66.203 (±12.742)	−1.7486	0.0889
Height (cm)	161.412 (±5.795)	161.294 (±6.974)	−0.1086	0.9142
BMI (Kg/m^2^)	27.413 (±4.772)	25.675 (±4.860)	−1.803	0.0808
Living status (alone, %)	8 (19.512)	15 (36.585)	2.9609	0.085
Unemployed (*n*, %)	25 (59.523)	19 (45.238)	1.718	0.190
Number of children	1.594 (±1.212)	1.486 (±1.017)	−0.426	0.672
Education (*n*, %)				
Primary	26 (61.904)	19 (46.341)	3.684	0.298
Secondary	2 (4.878)	6 (14.634)		
Professional	7 (16.666)	6 (14.634)		
University	7 (16.666)	10 (24.390)		
Years since diagnosis	9.027 (96% CI 6.980–11.074)	/	/	/
Medications (n, %)				
Anti-anxiety—bzd	28 (66.666)	5 (11.904)	26.403	0.000
Antihistaminic	3 (7.143)	3 (7.143)	0.0000	1.000
Gastro-intestinal Cardio-resp	15 (35.714)	8 (19.047)	2.9337 0.0737	0.087 0.786
	9 (21.428)	8 (19.047)		
Antidepressant Antidol/Antinf	18 (42.857)	2 (4.762)	16.800 19.012	0.000 0.000
	24 (57.143)	5 (11.905)		
Antidol/Opioid	17 (40.476)	0 (0)	21.313	0.000
BDI	22.444 (±6.143)	7.083 (±10.777)	−8.323	0.000
BDI cat (no depression)	26.316	81.578	23.356	0.000
BDI cat (depression)	73.684	18.421		
PSQI	13.839 (±4.754)	7.516 (±5.208)	−6.138	0.000
PSQI cat (no sleep impairment)	48.649	85.294	10.633	0.001
PSQI cat (sleep impairment)	51.351	14.706		
VAS pain	7.589 (±1.880)	1.875 (±1.914)	−12.259	0.000
S-FIQ	67.628 (±17.866)	16.466 (±15.819)	11.402	0.000
WPI	13.214 (±3.220)	2.714 (±1.979)	18.811	0.000
SSS	9.214 (±1.732)	3.119 (±2.491)	13.375	0.000

**Table 3:** DMRs test output (Metilene software). Chromosome coordinates related regions identified by Metilene with at least 1% difference in DNA methylation level, q values (calculated applying Bonferroni correction on 2DKS *p* values), CpG island number of the region, *p* values related to both MWU and 2DKS tests, mean methylation level HCs and FM patients are reported.

Gene	Chr (Start-Stop)	q-Value	#CpGs	*p* (MWU)	*p* (2D KS)	Mean Methylation Level HCs	Mean Methylation Level FM
*GCSAML*	chr1 (247518380–247518621)	0.083	87	0	0.0069	0.15808	0.16839

**Table 4:** DMCs test output: cytosines in which a significant difference in methylation levels has been found using the MWU test. Chromosome coordinates, q values (calculated applying Bonferroni correction on MWU *p* values), *p* values related to MWU test, mean methylation level HCs, and FM patients are reported.

	Chr	Start	Stop	q-Value	Mean Methylation Difference	*p* (MWU)	Mean Methylation Level HCs	Mean Methylation Level FM
GCSAML	chr1	247518426	247518427	1	−0.089641	0.019	0.42298	0.5126
	chr1	247518439	247518440	1	−0.068266	0.048	0.48173	0.55
	chr1	247518466	247518467	1	−0.070761	0.036	0.52852	0.59929
	chr1	247518583	247518584	0.6	−0.010065	0.00057	0.00	0.010066
	chr1	247518586	247518587	8.10 × 10^−11^	0.015519	7.7 × 10^−14^	0.015519	0.00
DRD3	chr3	114178637	114178638	1	−0.035952	0.046	0.38833	0.42429
TRPA1	chr8	72075678	72075679	1	−0.017392	0.0079	0.022132	0.039524
	chr8	72076373	72076374	1	−0.024068	0.046	0.8811	0.90517
	chr8	72076406	72076407	1	−0.016058	0.0095	1.6667 × 10^−7^	0.016059
IL25	chr14	23372248	23372249	1	−0.038382	0.0083	0.083523	0.1219
OXT	chr20	3071336	3071337	1	0.037212	0.025	0.59888	0.56167
	chr20	3071460	3071461	1	−0.010518	0.04	0.013291	0.02381
	chr20	3071465	3071466	1	−0.015701	0.021	0.025489	0.04119
	chr20	3071466	3071467	1	−0.014197	0.027	0.031755	0.045952
	chr20	3071468	3071469	1	0.012619	0.014	0.064762	0.052143
MCF2	chrX	139692297	139692298	1	−0.051717	0.0097	0.2423	0.29402
	chrX	139692312	139692313	1	−0.046018	0.019	0.30653	0.35255

**Table 5:** Simultaneous influence of the indipendent variables (the level of methylation levels of the genome regions related to *SYT2*, *GCSAML*, *GRM2*, *DRD3*, *NR3C1*, *TRPA1*, *MIR129-2*, *IL25*, *TNFRSF13B*, *OXT*, *MCF2*, depression measured through BDI questionnaire, and the demographic grouped factors) on the risk to have FM. Logistic multivariate regression model—explanatory variables: mean methylation levels, BDI, Cluster Two Factors; dependent variable: FM.

Logistic Regression						
Dependent Variable: Fibromyalgia						
Variables in the Equation	Odds Ratio	Robust Std. Err.	z	*p* > |z|	[95% Conf. Interval]	
*SYT2*	4.919	6.950	1.13	0.260	0.308	78.461
*GCSAML*	1.038	0.162	0.24	0.808	0.765	1.410
*GRM2*	0.614	0.143	−2.09	0.037	0.388	0.971
*DRD3*	3.035	12.078	0.28	0.780	0.001	7413.646
*NR3C1*	0.006	0.020	−1.45	0.147	5.17 × 10^−6^	6.157
*TRPA1*	12.892	22.999	1.43	0.152	0.391	425.457
*MIR1292*	0.091	0.162	−1.35	0.178	0.003	2.984
*IL25*	2.279	3.888	0.48	0.629	0.080	64.565
*TNFRSF13B*	7.145	15.473	0.91	0.364	0.102	498.124
*OXT*	1.115	0.533	0.30	0.761	0.464	2.854
*MCF2*	1.162	0.238	0.73	0.465	0.777	1.736
Depression—BDI	1.366	0.108	3.95	0.000	1.170	1.594
Demographic factors—Cluster Two steps	2.209	2.061	0.85	0.396	0.355	13.757
_cons	3.09 × 10^−17^	8.07 × 10^−16^	−1.45	0.146	1.68 × 10^−39^	567324.4
				Number of obs. = 65		
Log pseudolikelihood = −19.74235				Wald chi2 (7) = 32.48		
				Prob > chi2 = 0.0020		
				Pseudo R2 = 0.5611		

## Data Availability

The data presented in this study are available in the Supplementary Material and on request from the corresponding author.

## Supplementary Materials

The following are available online at https://www.mdpi.com/article/10.3390/jcm10214992/s1, Table S1. Data collected for each female participant (C1–C42: healthy controls; P1–P42: Fibromyalgia patients): age, diagnosis code, Fibromyalgia Impact Questionnaire (FIQ), Widespread Pain Index (WPI), Symptom Severity scale (SSS), Fibromyalgia Severity (FS) score (the sum of the WPI and SSS), Visual Analog Scale (VAS) for main FM symptoms, Pittsburgh Sleep Quality Inventory (PSQI) and Beck Depression Inventory (BDI) scores. Average (AVER) and standard deviation (STDEV) are reported for the collected data., Table S2: Differentially methylated Cytosines (DMCs) test (Metilene output), including both significant and not significant *p* values: Metilene output reveals DNA methylation mean scores for patients (42 FM women) and controls (42 related healthy sisters) measured in FM symptoms related regions. Figure S1: Study design., Figure S2: The sequence analyzed related to the GCSAML gene: the region is on the first exon in a CpG region of the gene that has multiple transcripts.
