# Supplementary material for: DNA Methylation Changes in Fibromyalgia Suggest the Role of the Immune-Inflammatory Response and Central Sensitization
Source: J Clin Med. 2021 Oct 27;10(21):4992. doi: 10.3390/jcm10214992 (PMC8584620; doi:10.3390/jcm10214992)
Supplement: Supplementary file 1 [file jcm-10-04992-s001.zip › jcm-1363278-supplementary.pdf]

Supplementary Figures

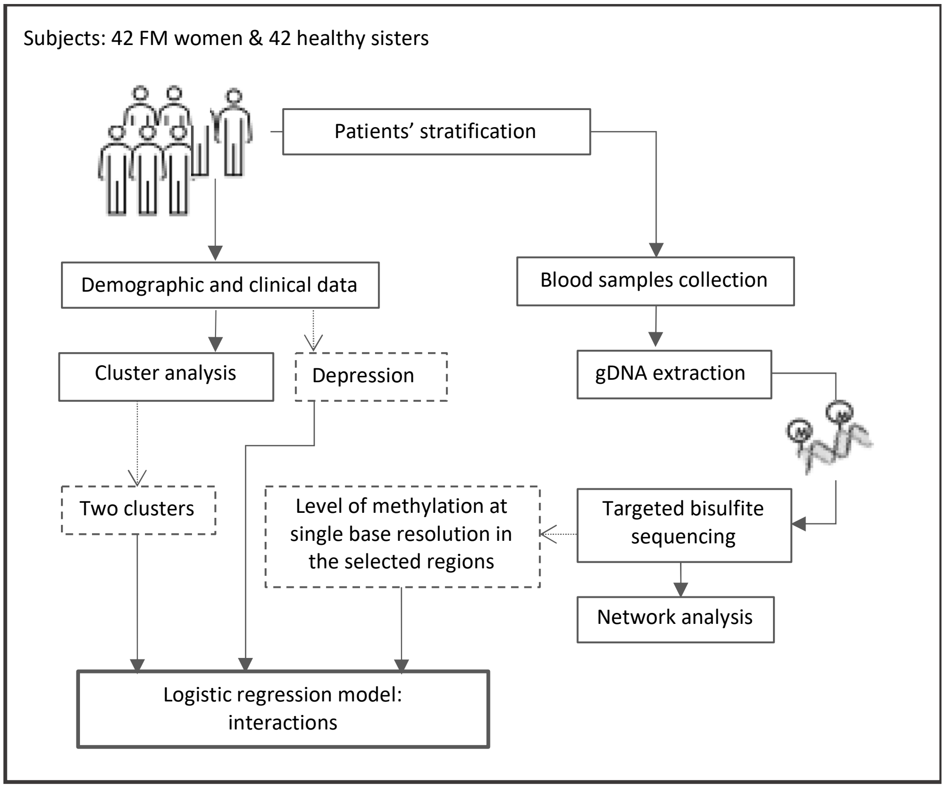

Figure S1. Study design.

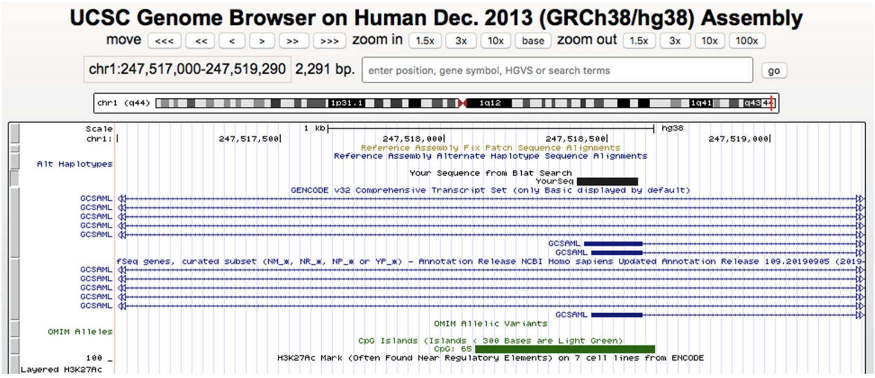

Figure S2. The sequence analyzed related to the *GCSAML* gene: the region is on the first exon in a CpG region of the gene that has multiple transcripts.

Supplementary Tables

Table S1. Data collected for each female participant (C1-C42: healthy controls; P1-P42: Fibromyalgia patients): age, diagnosis code, Fibromyalgia Impact Questionnaire (FIQ), Widespread Pain Index (WPI), Symptom Severity scale (SSS), Fibromyalgia Severity (FS) score (the sum of the WPI and SSS), Visual Analog Scale (VAS) for main FM symptoms, Pittsburgh Sleep Quality Inventory (PSQI) and Beck Depression Inventory (BDI) scores. Average (AVER) and standard deviation (STDEV) are reported for the collected data.

							VAS scales								
**CTRL**	**AGE**	**DIAG**	**S-FIQ TOTAL**	**WPI**	**SSS**	**FS**	**PAIN**	**HEALTH**	**RIGIDITY**	**FATIGUE**	**MOOD**	**HEAD ACHE**	**SLEEP**	**PSQI**	**BDI**
**C1**	39	1	28.720	2	7	9	1.782	2.376	1.782	2.376	3.762	0.297	4.455	10	19
**C2**	61	2	40.478	2	7	9	4.700	4.700	0.000	0.100	0.000	0.000	10.000	17	10
**C3**	48	1	7.000	6	3	9	1.400	1.100	1.400	1.300	1.100	1.100	4.000	12	2
**C4**	41	1		4	3	7									
**C5**	72	2	45.000	3	5	8	5.500	8.100	1.000	6.100	5.100	7.900	3.300	2	14
**C6**	44	1	7.000	2	2	4	1.400	1.200	5.100	4.500	2.200	0.100	1.000	6	1
**C7**	47	2	12.000	3	1	4	4.900	1.500	1.000	0.500	1.800	3.000	2.900	3	14
**C8**	51	1	6.529	1	0	1	0.200	0.200	0.300	0.200	0.400	0.300	0.200		5
**C9**		1	23.811	4	2	6	1.300	2.400	4.200	4.400	3.500	6.500	7.500		
**C10**	52	1	2.430	0	1	1	0.800	1.500	0.000	1.400	0.000	0.000	0.000	6	2
**C11**		1		3	6	9									
**C12**	40	1	20.430	4	6	10	2.500	2.300	1.500	4.900	3.000	2.400	8.400	16	10
**C13**	58	1	2.860	3	1	4	1.600	0.300	0.300	0.200	0.300	0.300	4.900	4	3
**C14**	33	1	7.000	2	1	3	0.300	0.000	0.000	0.700	0.000	0.700	7.600	11	5
**C15**	42	1	20.860	1	3	4	0.000	0.316	0.000	2.000	2.526	0.000	3.053	10	18
**C16**	44	1		0	3	3	0.211	0.947	0.000	0.211	0.737	0.105	0.526	7	12
**C17**	68	2	32.287	6	4	10	4.105	3.474	3.053	0.105	4.526	1.474	7.895	16	8
**C18**	44	1	3.430	2	2	4	0.211	0.211	0.211	0.105	0.105	0.105	0.211	4	2
**C19**	44	2	16.010	5	3	8	3.158	0.421	1.474	0.000	4.842	0.000	4.211	12	12
**C20**	59	2	46.720	3	8	11									
**C21**	39	1	11.816	2	5	7	1.474	2.737	0.105	4.421	4.526	6.842	2.632	3	6
**C22**	29	1	8.000	5	3	8	1.789	0.000	0.000	0.000	0.632	4.947	1.368		4
**C23**	53	2	63.768	3	5	8	6.737	6.632	4.316	4.421	4.421	2.737	5.895	13	12

**C24**	62	1	0.000	0	0	0	0.000	0.000	0.000	0.000	0.000	0.000	0.000	3	1
**C25**	34	1	9.990	0	0	0	0.000	0.000	0.000	0.000	0.000	1.765	4.471	3	0
**C26**	33	1	0.000	0	0	0	0.000	0.000	0.000	1.647	0.588	0.000	1.059	2	1
**C27**	38	1	36.010	3	1	4	2.235	2.471	0.235	0.824	6.471	0.706	2.588	9	18
**C28**	42	1	0.000	1	0	1	0.000	0.000	0.000	0.000	0.000	0.000	0.000	3	0
**C29**	45	1	0.999	1	1	2	0.235	1.176	0.235	0.706	0.706	0.706	0.824	2	0
**C30**	43	2	12.860	4	1	5	6.471	1.647	0.118	0.118	0.118	10.000	0.118	7	5
**C31**	54	1	9.997	5	8	13	0.000	0.000	0.000	0.000	0.000	0.000	10.000	6	9
**C32**	33	1	4.000	1	5	6	0.000	0.000	0.000	4.706	0.118	0.000	4.941	10	4
**C33**	28	1	20.092	0	4	4	0.000	5.176	0.706	4.941	4.824	4.706	9.176	16	19
**C34**	60	1	0.740	8	3	11	2.471	1.294	7.294	4.471	1.882	0.588	6.471		2
**C35**	38	1	13.430	4	6	10	1.882	0.824	0.000	6.118	0.000	0.000	5.294	12	4
**C36**	56	2	34.072	6	8	14	3.176	4.118	4.471	6.588	7.882	1.176	10.000	15	18
**C37**	56	2	33.340	5	1	6	3.176	3.647	8.941	3.529	1.176	1.176	0.471		5
**C38**	51	1	16.720	0	6	6	1.412	7.882	8.706	7.412	1.176	2.235	1.412	7	6
**C39**	57	1	0.000	3	2	5	0.471	0.353	0.353	0.471	2.353	2.471	0.471	2	4
**C40**	56	1	5.291	2	3	5	0.353	0.588	0.471	0.471	1.412	0.824	0.353	1	10
**C41**	53	1	15.860	3	1	4	1.882	2.000	2.706	2.824	0.941	0.353	0.353	4	5
**C42**	46	1	2.000	2	0	2	0.118	0.235	0.000	0.000	0.000	0.000	0.000	1	0
**AVER**	**47.325**		**15.937**	**2.714**	**3.119**	**5.833**	**1.742**	**1.842**	**1.538**	**2.122**	**1.875**	**1.680**	**3.540**	**7.500**	**7.105**
**STDEV**	**10.577**		**15.570**	**1.979**	**2.491**	**3.602**	**1.896**	**2.179**	**2.451**	**2.326**	**2.103**	**2.479**	**3.321**	**5.041**	**6.004**
**P1**	46	5	70.340	14	11	25	6.931	7.030	10.000	7.129	1.386	7.723	9.307	12	21
**P2**	65	5	51.314	12	11	23	6.500	4.400	9.500	5.500	5.200	4.800	8.900	20	
**P3**	47	5	93.815	17	11	28	8.700	9.800	9.700	9.800	9.800	6.900	9.700	15	30
**P4**	67	5	78.628	14	11	25	9.100	7.200	9.700	8.100	9.900	8.000	8.500		
**P5**	49	5	16.665	8	3	11	2.300	1.300	4.900	5.000	0.000	0.000	0.800	2	4
**P6**	47	5	77.322	13	10	23	6.200	6.700	3.000	8.400	8.500	3.600	4.900	16	21
**P7**	42	5	72.800	9	10	19	9.300	9.500	9.400	9.500	9.900	0.300	5.400	8	40
**P8**	53	5	85.345	13	10	23	9.300	9.900	9.800	10.000	2.700	1.000	10.000	9	26
**P9**	52	5	72.434	10	11	21	5.400	5.600	10.000	7.400	7.500	4.600	7.800		
**P10**	49	5	57.628	10	9	19	4.400	4.700	10.000	9.900	0.200	0.200	6.400	13	12

**P11**	58	5	93.487	17	9	26								17	32
**P12**	46	5	67.909	15	11	26	8.200	8.500	9.800	8.400	9.000	9.800	9.200	18	34
**P13**	54	5	43.284	10	8	18	4.700	4.800	7.000	8.000	2.300	1.300	1.000	8	9
**P14**	53	5		14	10	24									
**P15**	43	5	86.962	17	10	27	8.316	7.368	10.000	10.000	10.000	6.316	10.000	18	27
**P16**	39	5	80.342	19	10	29	8.737	8.737	7.053	9.158	9.158	3.053	9.263		38
**P17**	58	5	52.382	13	8	21	6.211	6.526	4.211	7.263	7.474	8.105	7.579	15	35
**P18**	60	5	62.343	19	8	27	8.316	5.368	5.684	5.789	6.947	5.474	7.895		26
**P19**	54	5	50.772	14	9	23	9.684	9.579	9.684	5.789	9.684	4.632	9.684	16	14
**P20**	61	5	50.160	11	6	17	4.947	5.263	8.105	5.158	3.053	1.895	5.579	10	9
**P21**	36	5	71.531	15	9	24	8.842	8.632	10.000	8.632	3.368	6.211	8.842	13	20
**P22**	75	5	64.105	15	9	24	8.000	2.000	9.789	7.579	3.684	4.105	9.368	16	10
**P23**	51	5	52.675	10	7	17	7.895	7.895	1.684	7.579	1.789	3.158	2.947	11	12
**P24**	60	5	92.346	17	10	27	10.000	10.000	10.000	10.000	10.000	10.000	10.000	21	38
**P25**	47	5	41.149	10	5	15	8.353	7.882	2.824	6.941	3.647	8.588	5.765	11	11
**P26**	22	5	75.918	13	8	21	9.647	9.647	2.588	9.765	0.235	10.000	10.000	18	12
**P27**		5	92.681	9	10	19	9.529	8.941	10.000	9.529	10.000	9.647	10.000	14	46
**P28**	49	5	37.337	6	8	14	4.588	7.882	2.353	4.588	3.412	3.412	1.176	8	16
**P29**	34	5	65.411	9	9	18	5.412	3.765	6.118	6.706	1.176	2.941	5.294	10	15
**P30**	43	5	74.531	16	11	27	8.471	8.353	9.647	8.941	5.294	8.706	6.000	11	21
**P31**	57		85.433	19	12	31	9.647	9.412	9.294	9.176	9.412	9.412	9.647	20	35
**P32**	45	5	73.336	16	11	27								20	44
**P33**	40	5	79.723	12	10	22	6.000	8.824	8.706	9.647	9.647	0.000	9.882	17	22
**P34**	55	5	59.010	11	8	19	6.706	7.059	10.000	10.000	6.941	3.059	3.176	12	12
**P35**	55	5	91.860	11	10	21	9.059	7.176	10.000	10.000	7.882	3.059	8.471	16	26
**P36**	46	5	54.672	15	8	23	7.059	7.647	7.647	7.647	4.118	5.176	4.941	7	18
**P37**	43	5	65.142	16	10	26	10.000	10.000	10.000	10.000	0.588	10.000	10.000	20	17
**P38**	44	5	94.347	15	10	25	9.647	9.647	9.412	7.765	8.471	5.294	9.294	19	29
**P39**	59	5	71.487	14	9	23	6.353	6.235	2.000	6.588	5.059	4.235	9.176	17	21
**P40**	58	5		11	9	20	6.941	3.529	6.118	7.882	5.647	1.294	6.706	11	16
**P41**	56	5	64.342	16	9	25	7.882	7.765	8.588	7.647	7.647	8.000	8.235	17	12
**P42**	56	5	83.746	10	9	19		10.000	10.000	10.000	10.000		10.000	7	18
**AVER**	**50.585**		**68.868**	**13.214**	**9.214**	**22.429**	**7.560**	**7.296**	**7.803**	**8.126**	**5.916**	**5.105**	**7.457**	**13.865**	**22.342**
**STDEV**	**9.685**		**17.817**	**3.220**	**1.733**	**4.289**	**1.896**	**2.285**	**2.808**	**1.629**	**3.437**	**3.208**	**2.757**	**4.632**	**10.826**

Table S2. Differentially methylated Cytosines (DMCs) test (Metilene output), including both significant and not significant p values: Metilene output reveals DNA methylation mean scores for patients (42 FM women) and controls (42 related healthy sisters) measured in FM related regions.

Chr	start	stop	q-value	mean methylation difference	#CpGs	pval (MWU)	pval (2D KS)	mean methyl control	mean methyl FM
chr1	247518407	247518408	1	-0.033329	1	0.44168	.	0.57261	0.60594
chr1	247518413	247518414	1	-0.064526	1	0.11537	.	0.57726	0.64178
chr1	247518422	247518423	1	-0.013254	1	0.60385	.	0.56487	0.57812
chr1	247518426	247518427	1	-0.067943	1	0.11537	.	0.47243	0.54038
chr1	247518435	247518436	1	-0.054567	1	0.25217	.	0.44734	0.50191
chr1	247518437	247518438	1	-0.036695	1	0.44701	.	0.29197	0.32867
chr1	247518439	247518440	1	-0.081927	1	0.085862	.	0.49216	0.57409
chr1	247518446	247518447	1	-0.065981	1	0.24848	.	0.47484	0.54083
chr1	247518454	247518455	1	0.014749	1	0.3295	.	0.017858	0.0031091
chr1	247518463	247518464	1	-0.045037	1	0.3295	.	0.57724	0.62228
chr1	247518478	247518479	1	-0.028706	1	0.74741	.	0.49835	0.52706
chr1	247518481	247518482	1	-0.027635	1	0.44168	.	0.55108	0.57872
chr1	247518498	247518499	1	0.015725	1	0.052222	.	0.016755	0.0010296
chr1	247518505	247518506	1	0.011128	1	0.1217	.	0.011128	2.38E-05
chr1	247518506	247518507	1	-0.011773	1	0.73389	.	0.59974	0.61152
chr1	247518520	247518521	1	0.014726	1	0.40542	.	0.017959	0.0032328
chr1	247518522	247518523	1	-0.014189	1	1	.	1.67E-03	0.01419
chr1	247518541	247518542	1	-0.068202	1	0.066663	.	0.57465	0.64285
chr1	247518550	247518551	1	0.020218	1	0.36151	.	0.66073	0.64051
chr1	247518553	247518554	1	0.015074	1	0.5549	.	0.026906	0.011832
chr1	247518554	247518555	1	-0.010049	1	0.77467	.	1.90E-03	0.010049
chr1	247518564	247518565	1	0.014635	1	0.29113	.	0.01883	0.0041948
chr1	247518566	247518567	1	-0.013171	1	0.95007	.	1.90E-03	0.013172
chr1	247518568	247518569	1	-0.052951	1	0.027755	.	0.66202	0.71497
chr3	51707199	51707200	1	-0.016213	1	0.48531	.	0.16117	0.17738
chr3	51707209	51707210	1	0.010693	1	0.66113	.	0.010693	1.67E-03
chr3	114178889	114178890	1	-0.026455	1	0.076507	.	0.11283	0.13929
chr3	114178938	114178939	1	-0.014882	1	0.025909	.	0.03726	0.052143
chr3	114179057	114179058	1	-0.018776	1	0.18254	.	0.095333	0.11411
chr8	72076045	72076046	1	0.016626	1	0.53705	.	0.51472	0.4981
chr11	4575858	4575859	1	0.019500	1	0.217	.	0.29905	0.27955
chr11	43581221	43581222	1	0.012045	1	0.021497	.	0.021129	0.0090842
chr11	43581242	43581243	1	0.013202	1	0.26346	.	0.020432	0.0072302
chr11	43581271	43581272	1	-0.015429	1	0.089178	.	0.012781	0.02821
chr11	43581296	43581297	1	-0.016972	1	0.049053	.	0.022165	0.039137
chr11	43581306	43581307	1	-0.011173	1	0.41559	.	0.0088797	0.020052
chr14	23372303	23372304	1	-0.010720	1	0.57303	.	0.87833	0.88905
chr17	16971852	16971853	1	-0.016130	1	0.11332	.	0.78101	0.79714

chr17	16972244	16972245	1	0.015841	1	0.23062	.	0.8706	0.85476
chr20	3071396	3071397	1	0.024708	1	0.51372	.	0.65995	0.63524
chr20	3071418	3071419	1	-0.028115	1	0.99286	.	0.0055438	0.033659
chr20	3071453	3071454	1	0.026037	1	0.32508	.	0.66122	0.63518
chr20	3071606	3071607	1	-0.039032	1	0.13056	.	0.58464	0.62367
chr20	3071615	3071616	1	0.026128	1	0.25589	.	0.79303	0.7669
chr20	3071627	3071628	1	-0.017024	1	0.5195	.	0.40155	0.41857
chr20	3071643	3071644	1	-0.018169	1	0.36623	.	0.41897	0.43714
chr20	3071649	3071650	1	-0.035845	1	0.33396	.	0.30344	0.33929
chr20	3071660	3071661	1	-0.018483	1	0.39044	.	0.17675	0.19524
chr20	3071672	3071673	1	-0.046248	1	0.1054	.	0.23066	0.2769
chr20	3071698	3071699	1	-0.021525	1	0.25966	.	0.23427	0.2558
chr20	3072579	3072580	1	0.026709	1	0.062775	.	0.83985	0.81314
chr20	3072581	3072582	1	-0.020724	1	0.35217	.	0.86304	0.88377
chr20	3072657	3072658	1	-0.019683	1	0.10349	.	0.65244	0.67213
chrX	139692245	139692246	1	-0.023783	1	0.72717	.	0.13689	0.16067
chrX	139692250	139692251	1	-0.011196	1	0.81607	.	0.26235	0.27355
chrX	139692253	139692254	1	-0.032995	1	0.44701	.	0.28068	0.31368
chrX	139692271	139692272	1	-0.013257	1	0.35682	.	0.0021758	0.015433
chrX	139692274	139692275	1	-0.067809	1	0.027128	.	0.45756	0.52536
chrX	139692285	139692286	1	-0.012456	1	0.82303	.	0.011681	0.024137
chrX	139692291	139692292	1	-0.027269	1	0.1093	.	0.019267	0.046536
chrX	139692297	139692298	1	-0.057321	1	0.11332	.	0.24152	0.29884
chrX	139692312	139692313	1	-0.056372	1	0.014961	.	0.3033	0.35967
chrX	139692315	139692316	1	-0.036593	1	0.13518	.	0.28335	0.31995
chrX	139692317	139692318	1	-0.044408	1	0.13753	.	0.34278	0.38719
chrX	139692321	139692322	1	-0.011993	1	0.57913	.	0.22928	0.24128
chrX	139692332	139692333	1	-0.029140	1	0.17963	.	0.37614	0.40528
